# Supplementary material for: Engineered repeat proteins as scaffolds to assemble multi-enzyme systems for efficient cell-free biosynthesis
Source: Nat Commun. 2023 May 4;14:2587. doi: 10.1038/s41467-023-38304-z (PMC10160029; doi:10.1038/s41467-023-38304-z)
Supplement: Supplementary file 1 — Supplementary Information [file 41467_2023_38304_MOESM1_ESM.pdf]

Supplementary Information

Engineered repeat proteins as scaffolds to assemble multi-enzyme systems for efficient cell-free biosynthesis

Alba Ledesma-Fernandez, Susana Velasco-Lozano, Javier Santiago-Arcos, Fernando López-Gallego,* and Aitziber L. Cortajarena*

Contents:

Supplementary Table 1. Table S1. ORF amino acid sequences of assembling elements: TRAP1-3 and TRAP2-3-1 scaffolds; and tagged-enzymes: FDH1, ωTA2 and AlaDH3 ………… 2

Supplementary Figure 1. Characterization of TRAP1-3 and TRAP2-3-1 scaffolds and tagged enzymes (FDH1, ωTA2 and AlaDH3) ………. 3

Supplementary Figure 2. Characterization of the two and three-enzyme assemblies based on biomolecular recognition by size exclusion chromatography using Superdex 200 (10/300) column ….… 4

Supplementary Table 2. Quantitative size analysis of the single component and scaffolded enzyme systems by SEC using a Superdex 200 (10/300) column ………… 4

Supplementary Table 3. Enzymatic activity of the enzymes upon peptide fusion …………… 5

Supplementary Figure 3. Michaelis-Menten curves of scaffolded enzyme systems ……….. 6

Supplementary Figure 4. Ratio of the reaction rate of free (Vf) (FDH1/AlaDH3) and scaffolded (Vs) (FDH1/AlaDH3@TRAP) enzyme systems as a function of NADH concentration, and Saturation plot as a function of NADH concentration ….... 7

Supplementary Table 4. L-Alanine conversion as a function of the NADH concentration for FDH1/AlaDH3 and FDH1/AlaDH@TRAP ….. 7

Supplementary Figure 5. Surface charge calculations of TRAP1-3 and molecular docking of TRAP1-3 scaffold and NAD^+/NADH cofactors ………… 8

Supplementary Figure 6. Fluorescence anisotropy-based binding assay of the NADH cofactor to the TRAP1-3 scaffold …....... 8

Supplementary Figure 7. L-Alanine synthesis in batch-mode ………….. 9

Supplementary Figure 8. Deuterated L-Alanine synthesis in batch-mode ………… 9

Supplementary Note 1. Solid-phase direct assembly immobilization on cobalt-agarose carrier ………….. 9

Supplementary Table 5. Immobilization parameters of sequentially co-immobilized FDH1/AlaDH3@TRAP on cobalt agarose (AG-Co^{2+}) and directly co-immobilized FDH1/AlaDH3@TRAP and FDH1/AlaDH3 on tri-functional carriers ….… 10

Supplementary Figure 9. Confocal fluorescence microscopy images (20X magnification) of co-immobilized FDH1/AlaDH3@TRAP on AG-Co^{2+} carrier 11

Supplementary Figure 10. Scheme of the tri-functional carrier 12

Supplementary Figure 11. Confocal fluorescence microscopy images (20X magnification) of co-immobilized FDH1/AlaDH3 on tri-functional carrier 12

Supplementary Table 6. Pearson Manders and co-localization coefficient determined through the analysis of confocal microscopy images displayed with FIJI software using JaCoP and co-localization colormap plugins 13

Supplementary Table 7. Specific productivity of L-Alanine (g x g_{enzyme}^{-1} x h^{-1}) for the biotransformation catalyzed by the soluble and immobilized systems 13

Supplementary Figure 12. Immobilization of free and scaffolded enzyme systems 14

Supplementary References 14

Results

Supplementary Table 1. ORF amino acid sequences of assembling elements: TRAP1-3 and TRAP2-3-1 scaffolds; and tagged-enzymes: FDH1, ωTA2 and AlaDH3. TRAP1 binding module in green, TRAP2 binding module in orange and TRAP3 binding module in purple. Cognate peptides 1 (MEEVV), 2 (MERVW) and 3 (MRRVW) directly fused to FDH, ωTA and LAlaDH enzymes in blue.

Assembly elements	ORF amino acid sequence
**TRAP1-3**	MGSSHHHHHHSSGLVPRGSHMGSALKEKELGNDAYKKKDFDTALKHYDKAKELDPTNMYILNQAAVYFEKGDYNKCRELCEKAIEVGRENREDYRLIAIAYARIGNSYFKEEKYKDAIHFYNKSLAEHRTPKVLKKCQQAEKILKEQGGSGLQALKEKELGNDAYKKKDFDTALKHYDKAKELDPTNMYIMNQAAVYFEKGDYNKCRELCEKAIEVGRENREDYRMIAYAYADIGDSYFKEEKYKDAIHFYNKSLAEHRTPKVLKKCQQAEKILKEQLE
**TRAP2-3-1**	MGSSHHHHHHSSGLVPRGSHMGSALKEKELGNDAYKKKDFDTALKHYDKAKELDPTNMYIMNQAAVYFEKGDYNKCRELCEKAIEVGRENREDYRMIAYAYARIGNSYFKEEKYKDAIHFYNKSLAEHRTPKVLKKCQQAEKILKEQGGSGLQALKEKELGNDAYKKKDFDTALKHYDKAKELDPTNMYIMNQAAVYFEKGDYNKCRELCEKAIEVGRENREDYRMIAYAYADIGDSYFKEEKYKDAIHFYNKSLAEHRTPKVLKKCQQAEKILKEQGGSGLQALKEKELGNDAYKKKDFDTALKHYDKAKELDPTNMYILNQAAVYFEKGDYNKCRELCEKAIEVGRENREDYRLIAIAYARIGNSYFKEEKYKDAIHFYNKSLAEHRTPKVLKKCQQAEKILKEQLE
**FDH1**	MGSSHHHHHHSSGLVPRGSHMEFKRSMKIVLVLYDAGKHAADEEKLYGCTENKLGIANWLKDQGHELITTSDKEGETSELDKHIPDADIIITTPFHPAYITKERLDKAKNLKLVVVAGVGSDHIDLDYINQTGKKISVLEVTGSNVVSVAEHVVMTMLVLVRNFVPAHEQIINHDWEVAAIAKDAYDIEGKTIATIGAGRIGYRVLERLLPFNPKELLYYDYQALPKEAEEKVGARRVENIEELVAQADIVTVNAPLHAGTKGLINKELLSKFKKGAWLVNTARGAICVAEDVAAALESGQLRGYGGDVWFPQPAPKDHPWRDMRNKYGAGNAMTPHYSGTTLDAQTRYAEGTKNILESFFTGKFDYRPQDIILLNGEYVTKAYGKHDKKKGSGSGSGSDDTSRMEEVV
**ωTA2**	MGSSHHHHHHSSGENLYFQGHMLRSNSNNKAWLKEHNTVHMMHPMQDPKALHEQRPLIIQSGKGVHITDVDGRRFIDCQGGLWCVNAGYGRREIIDAVTRQMEELAYYSLFPGSTNAPAIALSQKLTEVAAEEGMVKASFGLGGSDAVETALKIARQYWKLEGQPDKVKFVSLYNGYHGLNFGGMSACGGNAWKSSYEPLMPGFFQVESPHLYRNPFTNDPEELAEICAQILERQIEMQAPGTVAALIAEPIQGAGGVIVPPASYWPRLRQICDKYDILLIADEVITGLGRSGSLFGSRGWGVKPDIMCLAKGISSGYVPLSATLVNSRVARAWERDAGFTSVYMHGYTYSGHPVSCAAALAAIDIVLQENLAENARVVGDYFLEKLLILKDKHRAIGDVRGKGLMLAVELVKER

	ATKEPFGPADAYPLAISEACVNNGVMIRTIVNKLIISPPLTFTTEHVDEVIEVLDRAFVANP WKLGSGSGSDDTSRMERVW
**AlaDH3**	MGSSHHHHHHSSGLVPRGSHMEFKRSIIGVPKEIKNNENRVALTPGGVSQLISNGHRVL VETGAGLGSGFENEAYESAGAEIIADPKQVWDAEMVMKVKEPLPEEYVYFRKGLVLFTY LHLAAEPELAQALKDKGVTAIAYETVSEGRTLPLLTPMSEVAGRMAAQIGAQFLEKPKGG KGILLAGVPGVSRGKVTIIGGGVVGTNAAKMAVGLGADVTIIDLNADRLRQLDDIFGHQIKT LISNPVNIADAVAEADLLICAVLIPGAKAPTLVTEEMVKQMKPGSVIVDVAIDQGGIVETVD HITTHDQPTYEKHGVVHYAVANMPGAVPRTSTIALTNVTVPYALQIANKGAVKALADNTAL RAGLNTANGHVTYEAVARDLGYEYVPAEKALQDESSVAGAKLGSGSGSDDTSRMDDTS RMRRVW

a

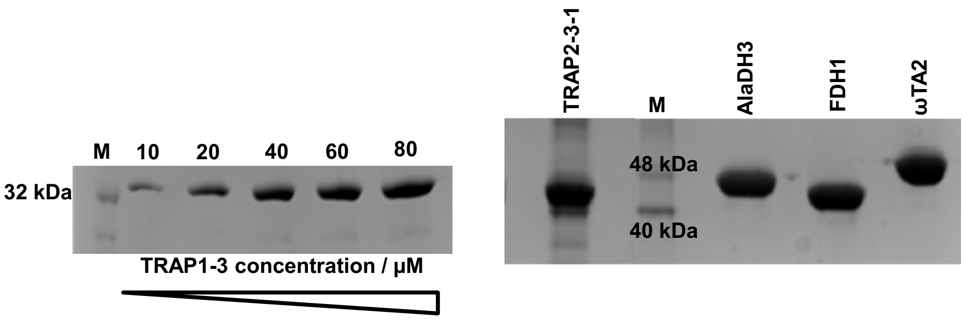

b

	TRAP1-3	FDH1	AlaDH3	TRAP2-3-1	ωTA2
Size by MALDI-TOF / kDa	32,621	45,137	44,323	47,932	50,822

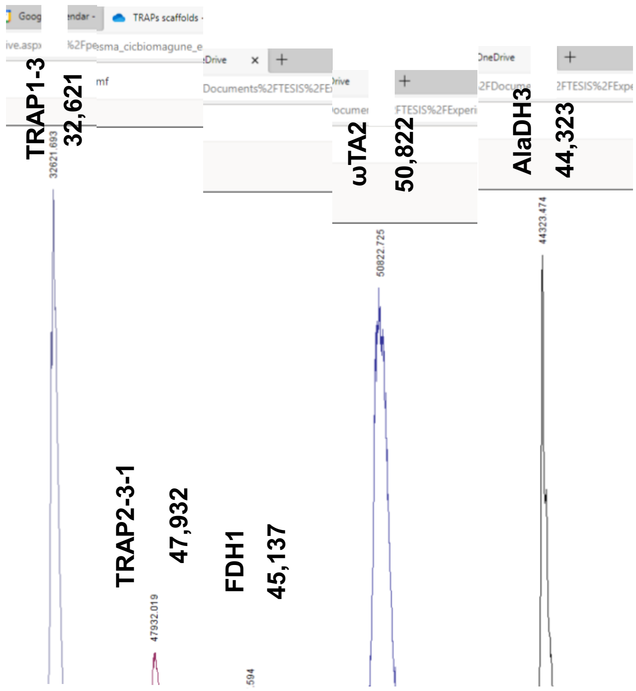

c

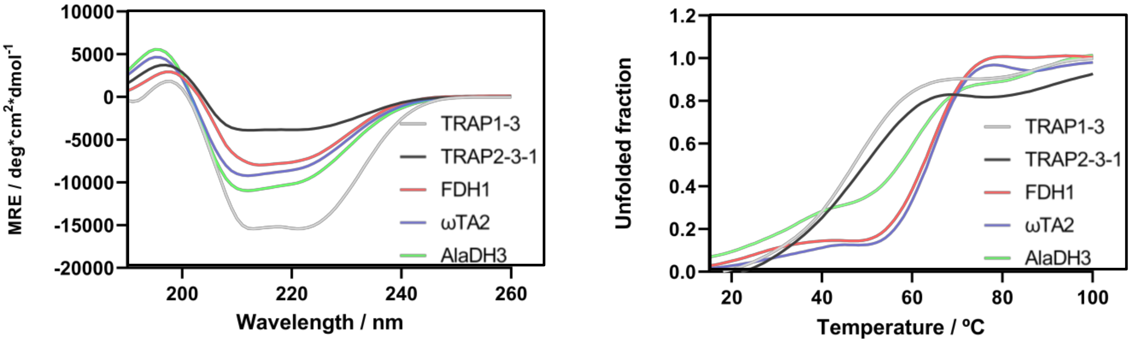

Supplementary Figure 1. Characterization of TRAP1-3 and TRAP2-3-1 scaffolds and tagged enzymes (FDH1, ωTA2 and AlaDH3). a) SDS-PAGE gel electrophoresis of purified TRAP scaffolds and tagged enzymes. Left panel: TRAP1-3 scaffold in different µM concentrations. Right panel: TRAP2-3-1 scaffold and tagged enzymes, AlaDH3, FDH1 and ωTA2. b) MALDI-TOF spectra and calculated masses from the MALDI spectra of the purified proteins. TRAP1-3 scaffold: 32,621 kDa, TRAP2-3-1 scaffold: 47,932 kDa, FDH1: 45,137 kDa, ωTA2: 50,822 kDa and AlaDH3: 44,323 kDa tagged enzymes. Masses calculated by the amino acid composition for TRAP1-3 scaffold: 32,559 kDa, TRAP2-3-1 scaffold: 47,803 kDa, FDH1: 45,276 kDa, ωTA2: 50,433 kDa and AlaDH3: 44,471 kDa tagged enzymes. c) Left panel, circular dichroism spectra of TRAP1-3 and TRAP2-3-1 scaffolds, FDH1, ωTA2 and AlaDH3 tagged enzymes. Right panel, thermal denaturation curves monitored by the decrease of the CD signal at 222 nm of TRAP1-3 and TRAP2-3-1 scaffolds, FDH1, ωTA2 and AlaDH3 tagged enzymes. TRAP1-3 in grey, TRAP2-3-1 in black, FDH1 in red, ωTA2 in dark blue, and AlaDH3 in green. Source data are provided as Source Data file.

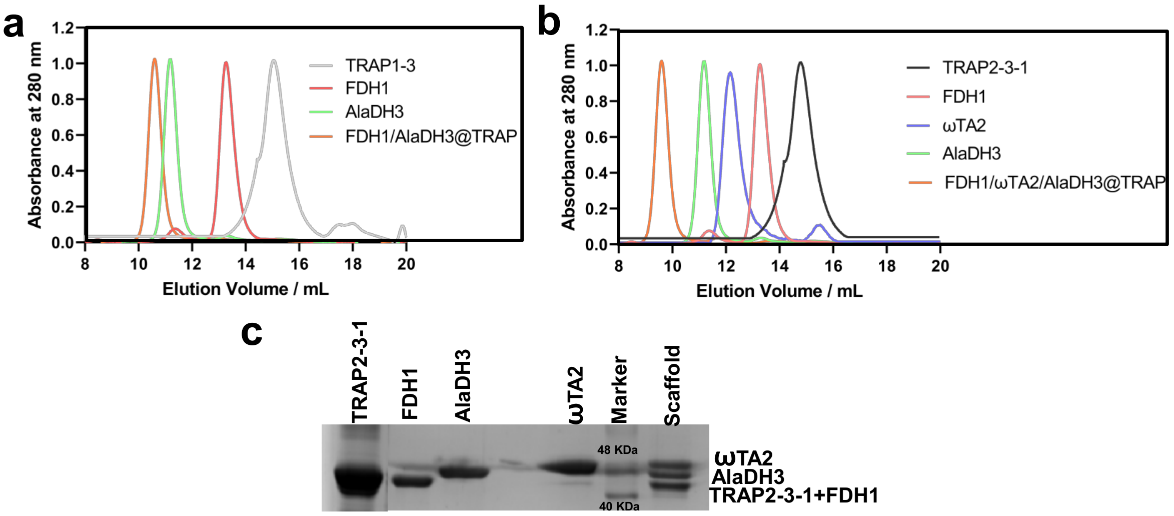

Supplementary Figure 2. Characterization of the two and three-enzyme assemblies based on biomolecular recognition by size exclusion chromatography (SEC) using Superdex 200 (10/300) column. a) SEC chromatogram monitored at 280 nm for TRAP1-3 scaffold, FDH1 and AlaDH3 tagged enzymes and the scaffolded enzyme system, FDH1/AlaDH3@TRAP. b) SEC chromatogram monitored at 280 nm for TRAP2-3-1 scaffold, FDH1, ωTA2 and AlaDH3 tagged enzymes and the scaffolded enzyme system, FDH1/ωTA2/AlaDH3@TRAP. c) SDS-PAGE gel of the purified elements after SEC analysis. From left to right: the free elements TRAP2-3-1, FDH1, AlaDH3 and ωTA2, protein marker and scaffold system FDH1/ωTA2/AlaDH3@TRAP. The FDH1/ωTA2/AlaDH3@TRAP composite elements were separated by size from largest to smallest: ωTA2, AlaDH3 and TRAP2-3-1+FDH1. The latter cannot be separated because their size in the gel is too similar. Source data are provided as Source Data file.

Supplementary Table 2. Quantitative size analysis of the single component and scaffolded enzyme systems by SEC using a Superdex 200 (10/300) column. The elution volumes for each TRAP protein, tagged enzyme, and scaffolded enzyme system are shown. The MW of the different systems was estimated from the elution volume by using a calibration curve (y = -0.213x + 7.816) generated with gel filtration calibration kit composed of a mixture

of well-defined proteins standards. The MW estimated experimentally is compared with that obtained from the amino acid sequence.

Sample	Elution volumen / mL	MW / kDa (by SEC)	MW / kDa (amino acid composition)
**TRAP1-3**	15.31	35	32
**TRAP2-3-1**	14.70	48	47
**FDH1 (dimer)**	11.21	95	90
**ωTA2 (tetramer)**	11.99	183	200
**AlaDH3 (hexamer)**	13.32	268	266
**FDH1AlaDH3@TRAP**	10.85	370	388 (1:1:1)
**FDH1/ωTA2/AlaDH3@TRAP**	9.55	605	603

Supplementary Table 3. Enzymatic activity of the enzymes upon peptide fusion. Specific enzymatic activities / U·mg^{-1} of the FDH, ωTA and LAlaDH enzymes compared to their corresponding tagged-enzymes, FDH1, ωTA2 and AlaDH3.

Enzymes	Specific enzymatic activity / U·mg^{-1}
**FDH**	0.82 ± 0.07
**FDH1**	0.74 ± 0.11
**ωTA**	5.58 ± 0.10
**ωTA2**	2.38 ± 0.05
**LAlaDH**	22.1 ± 0.01
**AlaDH3**	20.8 ± 0.33

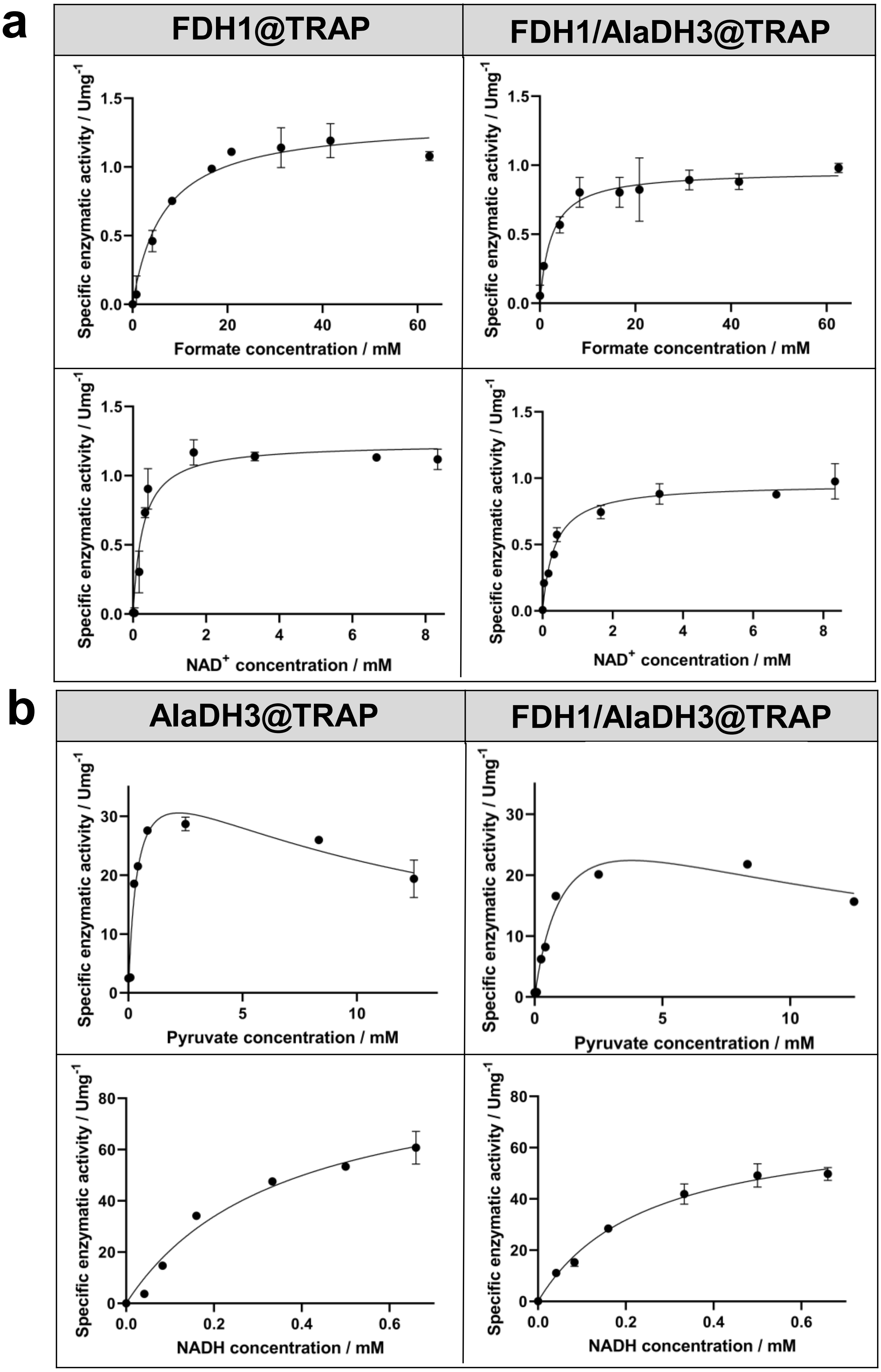
a
FDH1@TRAP
FDH1/AlaDH3@TRAP
Specific enzymatic activity / Umg^{-1}
Formate concentration / mM
NAD$^+$ concentration / mM
b
AlaDH3@TRAP
FDH1/AlaDH3@TRAP
Specific enzymatic activity / Umg^{-1}
Pyruvate concentration / mM
NADH concentration / mM

Supplementary Figure 3. **Michaelis-Menten curves of scaffolded enzyme systems.** A) Michaelis-Menten curves of the scaffolded enzyme systems (FDH1@TRAP and FDH1/AlaDH3@TRAP) using different substrates: variable formate concentration at 1 mM NAD^+ in 25 mM sodium phosphate buffer pH 7; and variable NAD^+ concentration at 100 mM formate in 25 mM sodium phosphate buffer pH 7. B) Michaelis-Menten curves of the scaffolded enzyme systems (AlaDH3@TRAP and FDH1/AlaDH3@TRAP) using different substrates: variable pyruvate concentration at 500 mM ammonium chloride, 0.5 mM NADH in 25 mM potassium phosphate buffer pH 8; and variable NADH concentration at 75 mM pyruvate, 500 mM ammonium chloride in 25 mM potassium phosphate buffer pH 8. Pyruvate kinetics were adjusted to a substrate inhibition model. The data are presented as the mean of three replicate experiments (n = 3), and error bars represent standard deviations. Source data are provided as Source Data file.

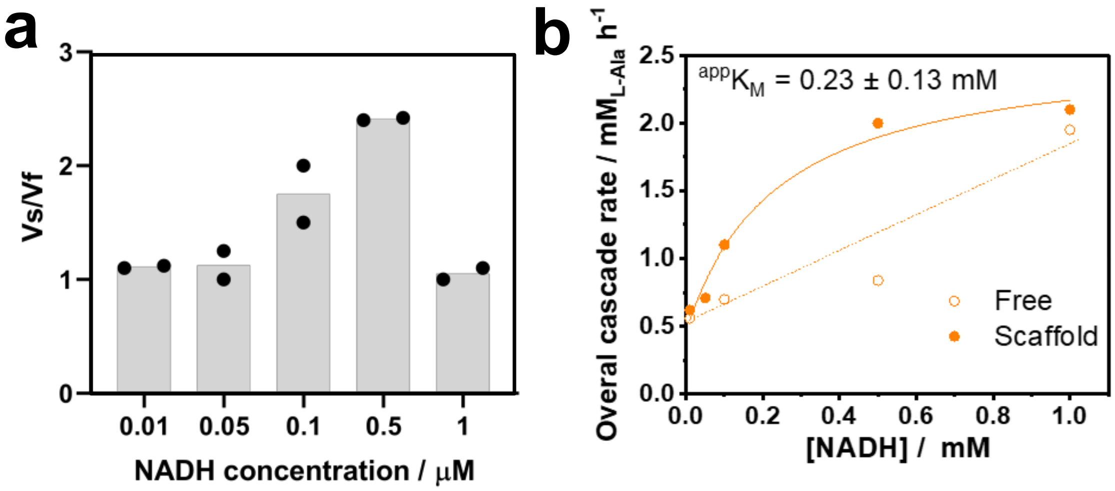

Supporting Figure 4. a) Ratio of the reaction rate of free (Vf) (FDH1/AlaDH3) and scaffolded (Vs) (FDH1/AlaDH3@TRAP) enzyme systems as a function of NADH concentration. Range of NADH concentrations evaluated: 0.01 mM, 0.05 mM, 0.1 mM, 0.5 mM, and 1 mM at an end point of 4 h. **b) Saturation plot of the overall enzyme cascade at different NADH bulk concentrations.** The apparent K_M value indicated within the plot refers to the apparent K_M of the cascade towards NADH upon fitting the rate datapoints to the Michaelis-Menten equation. The data are presented as the mean of two replicate experiments (n = 2). Source data are provided as Source Data file.

Supplementary Table 4. L-Alanine conversion as a function of the NADH concentration for FDH1/AlaDH3 and FDH1/AlaDH3@TRAP after 24-hour reaction. The range of NADH cofactor analyzed was from 0.01 mM to saturated conditions; 1 mM.

	L-Alanine concentration / mM	
NADH concentrations / mM	FDH1/AlaDH3	FDH1/AlaDH3@TRAP
0.01	13.5	15.0
0.05	14.8	17.1
0.1	16.8	26.0
0.5	20.29	49.0
1	46.98	50.0

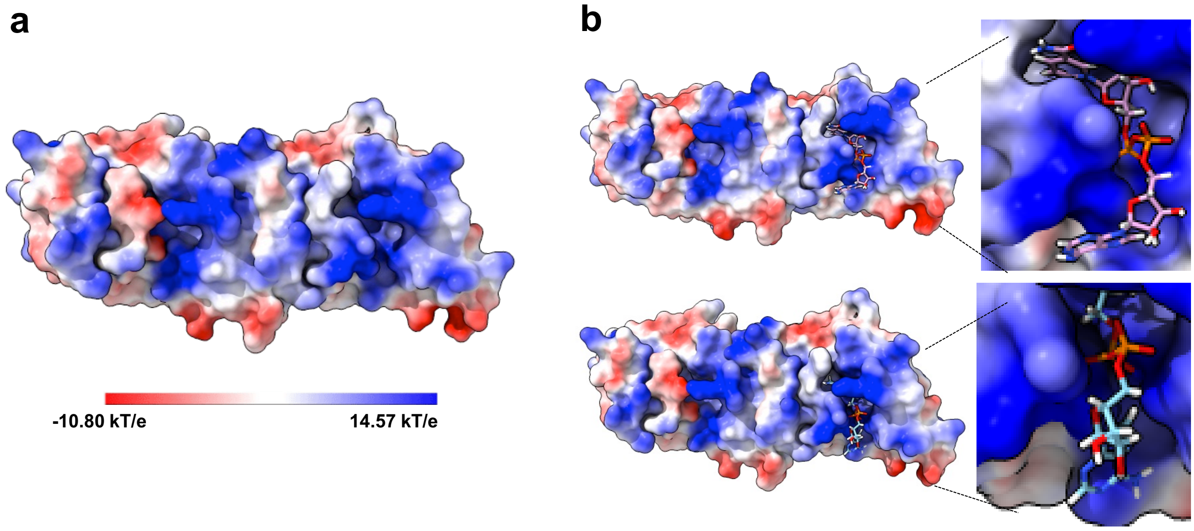

Supplementary Figure 5. Surface charge calculations of TRAP1-3 and molecular docking of TRAP1-3 scaffold and NAD$^+$/NADH cofactors. a) Electrostatic surface potential of TRAP1-3 protein scaffold colored according to calculated electrostatic potential of accessible surface area from −10.80 kT/e (red) to 14.57 kT/e (blue). b) Molecular docking studies on the scaffold-cofactor interaction. Molecular models for TRAP1-3 scaffold shown in surface charge representation, and NAD$^+$ (top) and NADH (bottom) cofactors shown in sticks representation in light pink and light blue, respectively. Both cofactors interact with the same specific positively charged pocket located in TRAP3 protein module. Source data are provided as Source Data file.

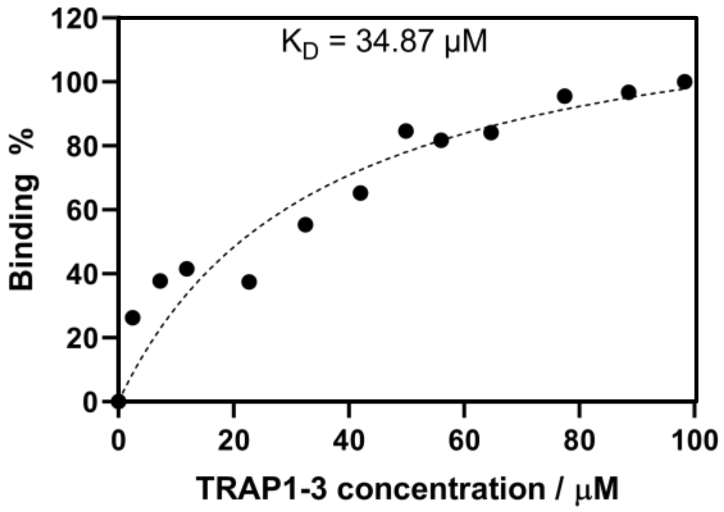

Supplementary Figure 6. Fluorescence anisotropy-based binding assay of the NADH cofactor to the TRAP1-3 scaffold. The binding curve shows the % binding of NADH to different concentrations of TRAP1-3 scaffold. The binding constant (K_D) of NADH respect to TRAP1-3 scaffold was 34.87 μM. The corresponding binding % of NADH to TRAP1-3 scaffold was fit to One Site-Specific binding model: y = 132.6*x/(34.87 + x) with and R-squared of 0.9158. Source data are provided as Source Data file.

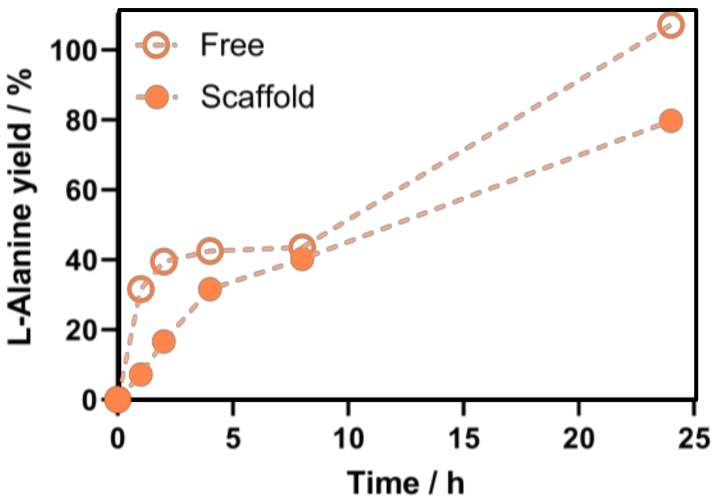

Supplementary Figure 7. L-Alanine synthesis in batch-mode. L-Alanine yield at 8:1 enzyme ratio of FDH1:AlaDH3 for the free enzyme system (FDH1/AlaDH3) and for the scaffolded enzyme system (FDH1/AlaDH3@TRAP). Reaction mixture: 100 mM formate, 75 mM pyruvate, 500 mM ammonium chloride, 0.5 mM NADH and 0.15 mM FAD^{+}. Source data are provided as Source Data file.

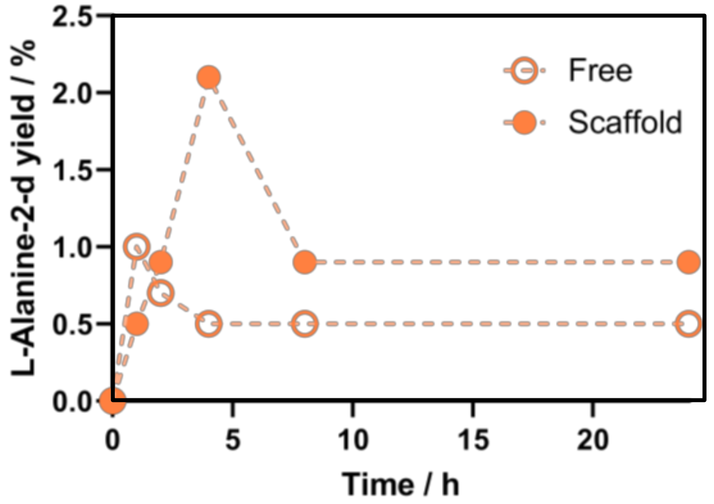

Supplementary Figure 8. Deuterated L-Alanine synthesis in batch-mode. Deuterated L-Alanine yield % at 1:1 enzyme ratio of FDH1:AlaDH3 for the free enzyme system (FDH1/AlaDH3) and for the scaffolded enzyme system (FDH1/AlaDH3@TRAP). Reaction mixture: 100 mM deuterated formate, 75 mM pyruvate, 500 mM ammonium chloride, 0.5 mM NADH and 0.15 mM FAD^{+}. Source data are provided as Source Data file.

Supplementary Note 1. Solid-phase direct assembly immobilization on cobalt-agarose carrier

As model solid support for the scaffold immobilization, we selected agarose porous microbeads functionalized with cobalt-chelates on which the His-tagged scaffoldin TRAP1-3 (His-TRAP1-3) is selectively bound. First, we immobilized the His-TRAP1-3 on the agarose microbeads, achieving a protein load of 6.5 $mg_{His\text{-}TRAP1,3}$ x $g_{carrier}^{-1}$, which means a concentration of scaffold per volume of solid support of 0.14 mM. Then, we tested if agarose microbeads primed with the His-TRAP1-3 were capable of orderly assembling FDH1 and

AlaDH3 on the solid phase. We follow a stepwise strategy similar to the one recently reported for the solid-phase assembly of cellulosome-based enzymatic scaffold [1]. Through mass balance between the offered solution and the supernatant upon the immobilization process, we calculated the load and the immobilization yield of each assembled enzyme. To quantify these parameters more accurately, we labeled the enzymes with fluorescent probes as follows, FDH1 with AlexaFluor-647 (AF647), and AlaDH3 with AlexaFluor-488 (AF488). Spectrophotometric quantification of the supernatants upon vacuum filtration informed us about the non-immobilized fraction of each enzyme, so we could indirectly calculate their bound fractions. In summary, the load of assembled FDH1 and AlaDH3 per mass of carrier was 4.52 mg x g^{-1} and 4.45 mg x g^{-1}, respectively (Supplementary Table 5). Accounting for loads of each element forming the scaffold, we obtained a scaffold with a molar ratio of 1:0.34:0.34, TRAP1-3:FDH1:AlaDH3. Hence, solid-phase assembly is less efficient than its counterpart in solution, which presented a molar ratio of 1:1:1. This result indicates that when two enzymes are assembled on an immobilized scaffolding, the assembly is impaired by potential steric hindrances. The impaired assembly of FDH1 and AlaDH3 in solid phase was supported by lack of submicrometric co-localization found in the confocal laser scanning microscopy (CLSM) analysis (Supplementary Figure 9) [2].

Supplementary Table 5. Immobilization parameters of sequentially co-immobilized FDH1/AlaDH3@TRAP on cobalt agarose (AG-Co^{2+}) and directly co-immobilized FDH1/AlaDH3@TRAP and FDH1/AlaDH3 on tri-functional carriers. Amount of protein added to 1 g of the two carriers for the immobilization process. Amount of protein loaded on 1 g of the two carriers after the immobilization process. Immobilization yield, Ψ = (added protein/loaded protein on the different carriers) x 100.

Assembly element	Added protein / mg/g	Loaded protein on AG-Co^{2+}carrier / mg/g	Ψ / %
**TRAP1-3**	6.5	6.5	100
**FDH1**	4.52	1.54	34
**AlaDH3**	4.45	1.51	34
**Enzyme system**	**Added protein / mg/g**	**Loaded protein on tri-functional carrier / mg/g**	**Ψ / %**
**FDH1/AlaDH3@TRAP1-3**	4.52/4.45@6.5	2.85/2.80@4.09	63
**FDH1/AlaDH3**	4.52/4.45	2.85/2.80	75

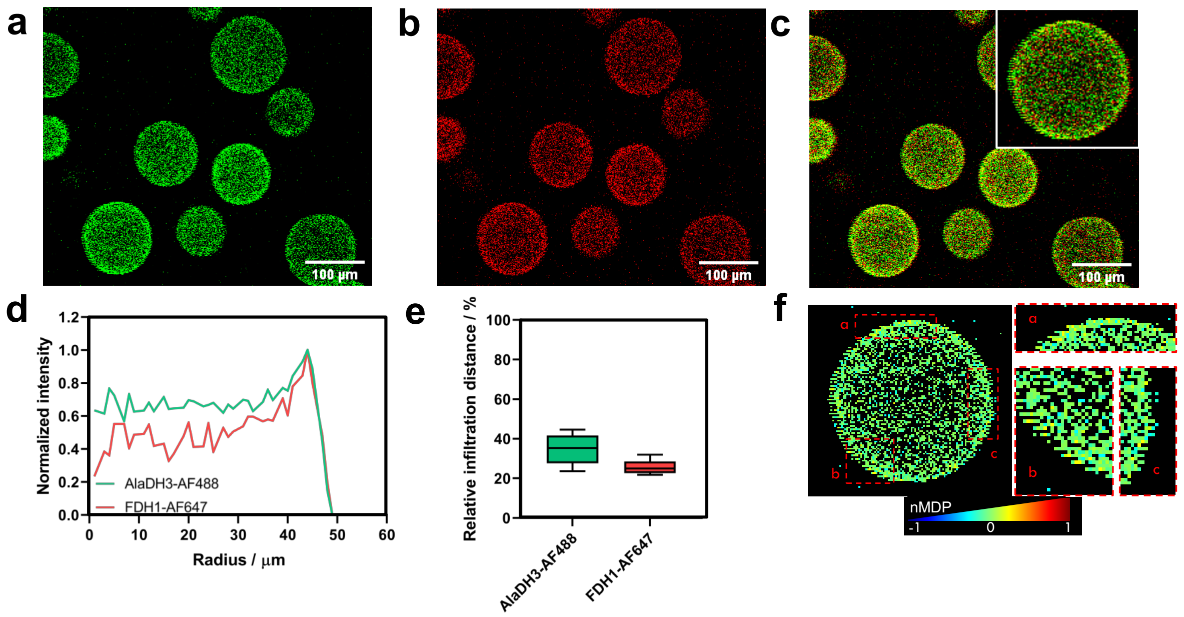

Supplementary Figure 9. Confocal fluorescence microscopy images (20X magnification) of co-immobilized FDH1/AlaDH3@TRAP on AG-Co^{2+} carrier. AlaDH3 and FDH1 were labeled with Alexa Fluor-488 (AF488) and Alexa-Fluor 647 (AF647). a) Spatial distribution of AlaDH3 (green channel), b) FDH1 (red channel) and c) overlay of the two fluorophores. The inlet shows a digital zoom of the micrograph of c. d) Radial profile and e) relative infiltration distance of labeled FDH1 and AlaDH3. Relative infiltration distance is defined as the fraction of the radius where the fluorescence intensity was higher than 50 % of the maximum intensity. The data are presented as the mean of six replicate experiments (n = 6), and error bars represent standard deviations. f) Co-localization map created with Colormap Image J plugin. The scale refers to the co-localization degree of the two labelled enzymes. Blue pixels (value = -1) mean the absence of co-localization. Red pixels (value = 1) mean a high degree of co-localization. The regions framed with the dashed red square represent those pixels with co-localization values > 0.1 where the scaffolded has been assembled as the spatial colocalization of the two fluorophores is statistically significant. Source data are provided as Source Data file.

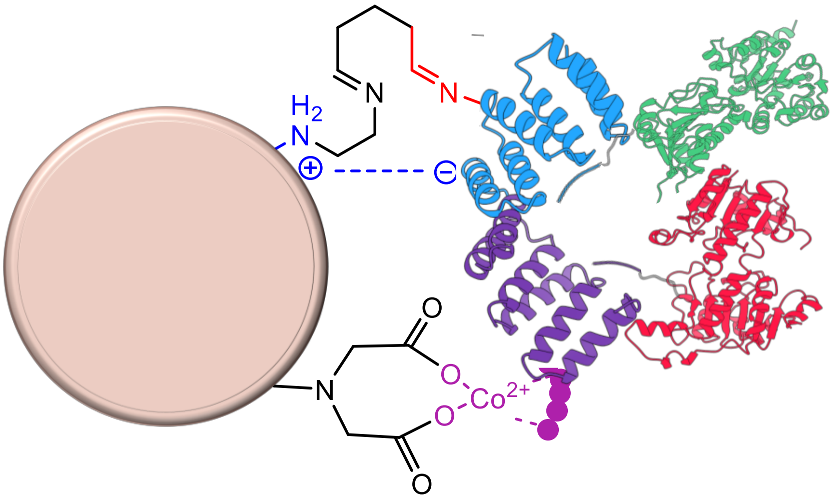

Supplementary Figure 10. Scheme of the tri-functional carrier. The tri functionality is due to the presence of cobalt chelates, positively charged amine groups and aldehydes at its surface.

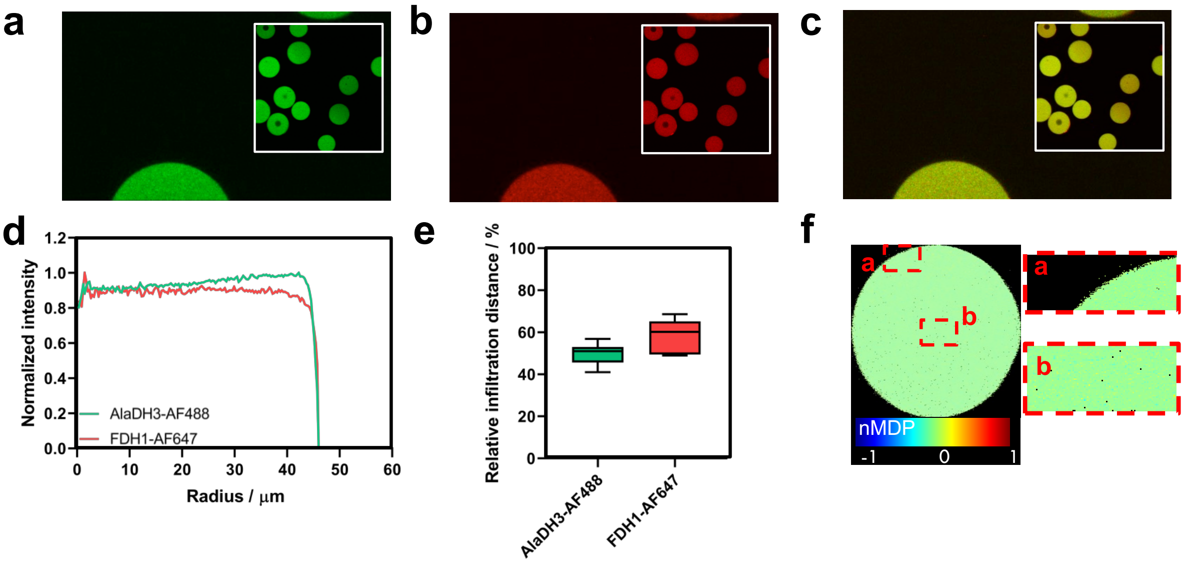

Supplementary Figure 11. Confocal fluorescence microscopy images (20X magnification) of co-immobilized FDH1/AlaDH3 on tri-functional carrier. AlaDH3 and FDH1 were labeled with Alexa Fluor-488 (AF488) and Alexa-Fluor 647 (AF647). a) Spatial distribution of AlaDH3 (green channel), b) FDH1 (red channel) and c) overlay of the two fluorophores. The inlet shows a digital zoom of the micrographs. d) Radial profile and e) relative infiltration distance of labeled AlaDH3 and FDH1. Relative infiltration distance is defined as the fraction of the radius where the fluorescence intensity was higher than 50 % of the maximum intensity. The data are presented as the mean of ten replicate experiments (n = 10), and error bars represent standard deviations. f) Co-localization map created with Colormap Image J plugin. The scale refers to the co-localization degree of the two labelled enzymes. Blue pixels (value = -1) mean the absence of co-localization. Red pixels (value = 1) mean a high degree of co-localization. The regions framed with the dashed red square represent those pixels with co-localization values > 0.1 where the spatial colocalization of the two fluorophores is statistically significant. Source data are provided as Source Data file.

Supplementary Table 6. Pearson Manders and co-localization coefficient determined through the analysis of confocal microscopy images displayed with FIJI software using JaCoP and co-localization colormap plugins. Pearson coefficient expresses the intensity correlation of fluorescence in two images. Manders coefficient A (MA) informs about the proportion of AF647-labeled FDH1 that overlaps in the space with the AF488-labeled AlaDH3, while Mander coefficient B (MB) reflects the opposite, *i.e.* the proportion of AF488-labeled AlaDH3 that overlaps in the space with the AF647-labeled FDH1. Index of correlation informs about the same as Pearson coefficient, indicating the distribution of the regions where pixels of both enzymes are present. The standard deviation was calculated based on the measurements on the 5 beads. System 1 is the solid-phase assembly of the scaffold using His-TRAP1-3 as priming unit previously immobilized on AG-Co^{2+}. System 2 is the scaffold assembled in solution and subsequently immobilized on the trifunctional agarose-based carrier activated with aldehydes, cobalt chelates and positively charged amine groups. System 3 is the benchmarked system where non-scaffolded AlaDH3 and FDH1 are immobilized on the tri-functional carrier.

System	*Coef.* Pearson	*Coef.* MA	*Coef.* MB	Index of correlation
1	0.29 ± 0.05	0.39 ± 0.03	0.14 ± 0.02	0.40 ± 0.03
2	0.82 ± 0.05	1 ± 0	0.99 ± 0	0.58 ± 0.04
3	0.85 ± 0.04	0.99 ± 0	0.93 ± 0.07	0.55 ± 0.04

Supplementary Table 7. Specific productivity of L-Alanine (g x $g_{enzyme}{}^{-1}$ x h^{-1}) for the biotransformation catalyzed by the soluble and immobilized systems.

Formulation	FDH1/AlaDH3	FDH1/AlaDH3@TRAP
Soluble	0.92	5.21
Immobilized	0.98	1.11

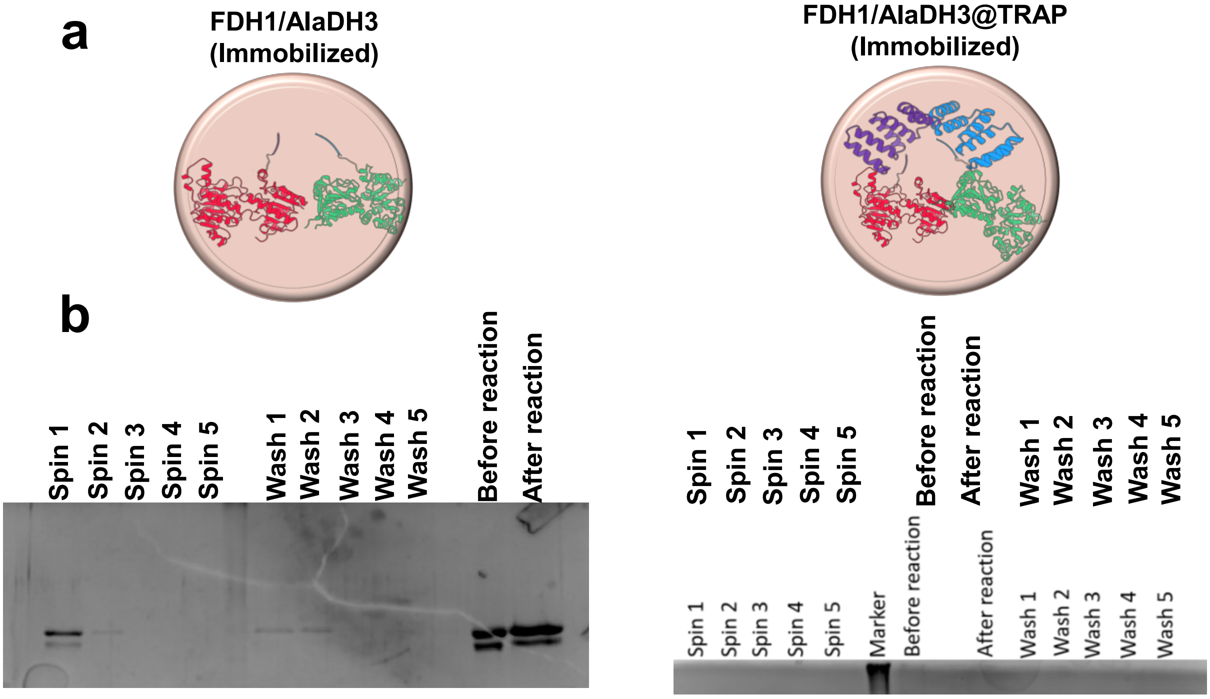

Supplementary Figure 12. Immobilization of free and scaffolded enzyme systems. a) Scheme of the immobilized systems analyzed in the assay: FDH1/AlaDH3 and FDH1/AlaDH3@TRAP. b) SDS-PAGE gel electrophoresis of FDH1 (44 kDa band) and AlaDH3 (48 kDa band) enzymes before and after performed the reusability test. Spin samples are the reaction crude after 24 h. Wash samples were the wash of the heterogeneous biocatalysts after each cycle with 10 volumes of reaction buffer. The spin and wash steps were shown in order to follow the process. Left panel: reusability test of free enzyme system, FDH1/AlaDH3. Right panel: reusability test of scaffolded enzyme system, FDH1/AlaDH3@TRAP.

Supplementary References

1. Zeballos, N., Diamanti, E., Benítez-Mateos, A. I., Schmidt-Dannert, C. & López-Gallego, F. Solid-Phase Assembly of Multienzyme Systems into Artificial Cellulosomes. *Bioconjug. Chem.* **32**, 1966–1972 (2021).
2. Rai, V. & Dey, N. The Basics of Confocal Microscopy. *Laser Scanning, Theory Appl.* 3–5 (2011).
